# Looking deeper: does a connection exist between fatigue and attentional deficits in Parkinson's disease? A conceptual framework

**DOI:** 10.3389/fneur.2023.1212876

**Published:** 2023-08-11

**Authors:** Paola Ortelli, Viviana Versace, Leopold Saltuari, Anna Randi, Jakob Stolz, Sabrina Dezi, Roberto Maestri, Susanne Buechner, Nir Giladi, Antonio Oliviero, Luca Sebastianelli, Davide Ferrazzoli

**Affiliations:** ^1^Psychology Service, Hospital of Bressanone (SABES-ASDAA) - Teaching Hospital of the Paracelsus Medical Private University (PMU), Bressanone-Brixen, Italy; ^2^Department of Neurorehabilitation, Hospital of Vipiteno (SABES-ASDAA), Vipiteno-Sterzing, Italy - Teaching Hospital of the Paracelsus Medical Private University (PMU), Salzburg, Austria; ^3^Istituti Clinici Scientifici Maugeri, IRCCS, Department of Biomedical Engineering of Montescano Institute, Pavia, Italy; ^4^Department of Neurology, Hospital of Bolzano (SABES-ASDAA) - Teaching Hospital of the Paracelsus Medical Private University (PMU), Bolzano, Italy; ^5^Brain Institute, Tel Aviv Soursky Medical Center, Tel Aviv, Israel; ^6^Faculty of Medicine and Sagol School of Neurosciences, Tel Aviv University, Tel Aviv, Israel; ^7^FENNSI Group, Hospital Nacional de Parapléjicos, Servicio de Salud de Castilla La Mancha, Toledo, Spain

**Keywords:** fatigue, attention, Parkinson's disease, intra-individual variability, TMS, frontal lobe

## Introduction

Parkinson's disease (PD) is a chronic and progressive neurodegenerative disorder characterized by asymmetrical limb bradykinesia, rigidity and tremor. A *plethora* of cognitive, emotional and vegetative non-motor symptoms are also frequent and can greatly impact on daily living (1). Among them, fatigue is estimated to occur in about 50% of patients with PD (2–4). Even more important, one-third of these subjects report this symptom as one of the most disabling in terms of quality of life (5). Friedman et al. (3) proposed to define PD fatigue as a *sense of exhaustion unexplained by drug effects, other medical, or psychiatric disorders, present for a defined period, and associated with other fatigue-related symptoms, such as reduced motivation and non-restorative rest, or constraints* (6). There are neither established empirical approaches to the treatment of fatigue in PD nor accepted pathophysiological mechanisms underlying this debilitating symptom (7–9). Based on several published studies (10–13), we hypothesize that fatigue in PD could be an indirect expression of attention deficit, a common cognitive disturbance in PD that can frequently be interpreted by the patient as fatigue. A recent report has linked attention deficits and fatigue in several neurological disorders, including PD (14). Studying intra-individual variability (IIV) in reaction times (RTs) and the use of transcranial magnetic stimulation (TMS) could have the power to shed light on the interaction between fatigue and attention. Together with a brief review of the proposed models for explaining fatigue in PD, we suggest why IIV and TMS could help in understanding this symptom and propose a framework for future studies.

## Fatigue in PD, the link with attention and study modalities

Fatigue in PD is not related to decreased force-generating capacity during voluntary muscular contractions but to an increased sense of effort in both motor and cognitive tasks (15, 16). Disruption of the reciprocal loop between the striatum, frontal and limbic structures, following dopamine depletion (17, 18), was shown to be associated with fatigue in PD (19, 20). Nevertheless, the effect of dopaminergic therapies on fatigue remains unclear (21–23) and it is actually known that other non-dopaminergic networks are involved in fatigue generation (24). Coherently, it has been reported that the presence of fatigue is associated with serotoninergic denervation in the basal ganglia (BG) and limbic circuits (12). These changes could disrupt the integrity of different motor-cognitive processes (12), leading to a dissociation between motivation to act and motor execution, which could finally result in reluctance to move and feeling of fatigue (25). Because of performing movements is a decision-making process and the choice to move is taken considering the effort necessary to reach the goal (26), pathological fatigue could emerge from deficient evaluation of internal (somatic) input associated with abnormal feedback of perceived exertion (27). Accordingly, clinical and experimental evidence suggest that fatigued PD patients present in decision-making processes (28). The orbitofrontal cortex (OFC) is implicated in decision-making process, as well as in emotion regulation and reward processing. A strong contribution to the process of decision-making is provided by the dorsolateral prefrontal cortex (DLPFC), that is mainly involved in executive functions and is associated with attention to the selection of action (29). Despite different relations have been observed between fatigue and cognitive, motivational and emotional problems (in terms of depression, episodic anxiety, cognitive apathy, sleepiness, and subjective memory impairment) (13, 30), this symptom has been found to affect also non-depressed and non-demented PD patients to the same extent (31). In a prospective, 8-year longitudinal study of 233 PD patients (32), fatigue was found to be persistent in more than half of the patients and the authors concluded that non-motor features, such as depression and excessive daytime sleepiness, cannot explain fatigue (32). Using resting-state functional MRI in drug-*naïve* patients with early PD, Tessitore et al. (10) found that fatigue is associated with decreased connectivity within the supplementary motor area and increased connectivity within the prefrontal and posterior cingulate hubs of the default mode network. In line with these data, it has been found that PD patients with fatigue manifest significantly lower executive network efficiency, lower accuracy and less efficient attentional-alerting network (11). Other relevant findings support a link between PD-related fatigue and attention-demanding motor tasks (33). Martino et al. (34) designed a protocol based on sequential finger opposition movements paced to a 2 Hz metronome signal and repeated continuously for 5 min (34). This motor task requires high attentional demand and both spatial and temporal accuracy (33, 34). The authors administered this finger sequential task to PD patients with and without fatigue and found that the accuracy of fatigued PD patients deteriorated more than in non-fatigued PD patients, and that change over time correlated significantly with the burden of subjective fatigue complaints. Interestingly, subjective fatigue complaints were not associated with performance deterioration on an internally paced (un-cued) version of the same task (33).

All these observations lead toward the hypothesis that fatigued PD patients could fail in initiating and maintaining attentional tasks that require self-motivation and/or manifest an inability to inhibit/control the occurrence of excessive or distracting internal and external stimuli (10, 25, 27). Despite the plausible link between attention and fatigue in patients with PD, the lack of extensive neuropsychological evaluations, together with the absence of any neurophysiological measures, does not allow to disentangle the complex interplay between fatigue and attention in PD and the pathophysiology of this symptom remains still largely unknown.

Classically, researchers referred on reaction times (RTs) tasks for exploring the link between “central” fatigue and attention. These studies highlighted that patients with fatigue manifest defective attention (35, 36) and present increased IIV in RTs tasks exploring executive attention (37). IIV consists of within-person fluctuations in cognitive performance and their (in-)stability across time. It is believed to reflect the brain activity at different neural level (37) as provides information about attentional/executive control demand (38, 39), thus representing a useful method for understanding the neurological dysfunctions (40–42). Low IIV (i.e., high consistency across scores) is hypothesized to reflect neurological integrity, whereas high IIV (i.e., low consistency across scores) could be indicative of neurological compromise (43). IIV has been found to be greater in PD patients relative to controls, both in global cognition and in attentive functions (41, 44–46). These findings are in line with the notion that deficits in attention present early in the disease course and are among the most frequent non-motor symptoms in PD (47).

Because of the progressive degeneration of the dopaminergic transmission in PD alters the direct pathway leading to the need for a massive exploitation of executive-attentive resources to express motor behaviors (48–55), the measure of IIV and inconsistency could be extremely relevant also as a motor-cognitive marker.

By referring to IIV analysis of RTs, different studies aimed to define the attentive impairment and its relation with fatigue in other neurological conditions. A recent study (56) assessed 74 post-COVID patients complaining of high levels of fatigue with computerized Sustained Attention and Stroop tasks. For studying IIV, RTs distributions of performances in computerized tasks were fitted with ex-Gaussian distribution. In sustained attention task, mean, μ, σ and τ values were significantly higher in patients with respect to controls (56). These findings strengthen the role of these measures for detecting links between perceived fatigue and attentive deficits in neurological patients.

The pathophysiology of neural pathways involved in fatigue and attention deficits could be further explored through TMS (57–65). In a typical TMS study, the researchers first determine the threshold required to activate a muscle. The threshold is typically defined as a stimulation intensity required to evoke a Motor Evoked Potential (MEP) of > 50 micronV recorded from the target muscle in five of ten trials. In normal subjects, intermittent submaximal exercise is accompanied by increase in TMS-evoked MEP amplitude during exercise before fatigue develops, whereas, after fatigue has developed, a decrease in MEP amplitude relative to baseline can be found. Both, post-exercise facilitation and post-exercise depression are most likely mediated by cortical mechanisms (66). Lou et al. (67) found that PD patients in “off” state have more pronounced post-exercise facilitation and absent post-exercise depression compared with normal controls. A small dose of levodopa/carbidopa (100/25 mg) reduced the MEP amplitudes. Therefore, the investigators concluded that dopamine may play a role in exacerbated physical fatigability in PD because levodopa/carbidopa normalized abnormal cortico-neural excitability in these patients (65, 67). The increased MEP amplitude and more pronounced post-exercise facilitation might represent compensatory mechanisms for reduced excitatory inputs from the premotor and the supplementary motor areas in PD (65, 67). These findings related to MEP measures do not completely explain the cognitive, attention-related aspects concerning fatigue and the contribution of other non-dopaminergic pathways in fatigue generation in PD. Nevertheless, the potential relevance of TMS in addressing the pathophysiology of fatigue in PD could be clearly understood when looking at the sequence effect (SE). SE is characterized by progressive slowness in speed or a decrease in amplitude of sequential movements and it represents a main feature of bradykinesia (68). It may be associated with altered cortical excitability: as the BG are important for planning movement amplitude, the aberrant output from the BG to the motor cortex may produce this abnormality (69). Different studies have followed the hypothesis that SE observed during the execution of complex movements may be related to fatigue (70, 71). Nevertheless, it remains still unclear whether fatigue correlates with these motor-behavioral abnormalities in PD or not. In this concern, Bologna and colleagues (72) investigated whether objective measures of bradykinesia (amplitude, speed and decrement of repetitive finger tapping) have any relationship with neurophysiological measures in primary motor cortex as assessed by means of TMS measures. PD patients tapped more slowly and with smaller amplitude than normal, and displayed decrement as tapping progressed. They also had steeper input/output curves, reduced short-interval intracortical inhibition and a reduced response to the paired associative stimulation protocol. Further, bradykinesia features correlated with the slope of the input/output curve and the after-effects of the paired associative stimulation protocol (72). These results suggest that a tight relation linking neurophysiological changes in primary motor cortex and bradykinesia exist. Therefore, because of SE (as a main feature of bradykinesia) and less efficient attentional-alerting network could be both related to some extent with perceived fatiguability, we could assume that different TMS measures, given their potential to study complex neural networks, may be useful to explore deeper the pathophysiology of fatigue in PD. As a matter of fact, TMS has been adopted to unveil fatigue-related mechanisms in different neurological conditions (57–65). In patients with multiple sclerosis, the Cortical Silent Period (CSP), an intracortical, mainly GABA_B_-mediated inhibitory phenomenon, was found to be shorter in patients than in controls (57). As fatigue developed, CSP changes involved both the “fatigued” and the “unfatigued” muscles, suggesting a cortical spread of central fatigue mechanisms. Interestingly, chronic therapy with amantadine annulled differences in CSP duration between controls and patients, possibly through restoration of more physiological levels of intracortical inhibition in the motor cortex (57). In a recent cross-sectional observational study (61) in 59 non-depressed stroke survivors suffering from non-exercise induced fatigue (PSF), the authors examined the relationship between inter-hemispheric inhibitory balance (IIB) of homolog neural populations and subjectively reported PSF severity (measured with Fatigue Severity Scale). The authors found an association between individuals' levels of IIB in M1 and the reported levels of persistent PSF (61). Interestingly, IIB has been previously linked properly with attentional and affective disorders (61). In patients suffering from long-lasting fatigue and/or cognitive difficulties after mild SARS-CoV-2 infection longer CSP, together with impairments in long-interval intracortical inhibition and short-latency afferent inhibition, was found, thus indicating altered GABA_B_-ergic and cholinergic neurotransmission (64).

## Study proposal and framework

We believe that literature data go through the idea that future studies putting together IIV in attentive RTs analysis and a wide TMS-based assessment could contribute to the knowledge of the intricate link between fatigue, motor behavior alterations and attention deficits in PD.

Starting from these assumptions, in the following section we will outline which clinical, IIV and TMS paradigms we intend to use in future studies for better understanding the pathophysiological mechanisms underlying fatigue in PD.

### Evaluation of fatigue

Presence of fatigue can be defined based on the 16-item Parkinson Fatigue Scale (PFS-16) (73), which was developed for use in routine clinical practice and has been recommended for screening and rating the severity of fatigue in PD taking into account possible overlapping with other motor and non-motor symptoms (74). PFS-16 provides a measure of fatigue, which is independent of affective, sleep and cognitive disturbances. Brown et al. (73), using the full Likert scale, found that an average score greater than 2.9 distinguishes those who experienced fatigue from those who did not with a sensitivity of 81.0% and specificity of 85.7%. Therefore, according with this finding and previous reports (11), a PFS-16 threshold of 2.9 can be adopted to define the presence of fatigue and to differentiate “fatigued PD patients” and “non-fatigued PD patients”.

### IIV in RTs

We intend to apply the study of IIV to the following computerized RTs tasks in fatigued and non-fatigued PD patients in order to unveil whether such aspects of mental-cognitive fatigue could be related to dysfunctions in attentional networks:

#### Sustained attention task

SAT evaluates the speed with which subjects respond to a specific environmental stimulus that are presented at randomized intervals. For example, patients have to press a response button as quickly as possible after the appearance, on the computer screen, of a target that disappear immediately after striking the response key (56, 75).

#### Stroop task

ST assesses to inhibit cognitive interference, which occurs when automated processing of a stimulus feature affects the simultaneous processing of another attribute of the same stimulus (76). The task can be divided into two conditions: word Color Naming (WCN), and Color Naming (CN). In this paradigm (56, 75), patients have to press corresponding keys related to differently colored circles (CN) as fast as possible. In WCN (“interference condition”) names of colors are printed in inconsistent colors, and subjects have to press a key corresponding to the color of the ink instead of the word's meaning. Thereafter, participants will have to perform a less automated task (naming ink color) while inhibiting the interference arising from a more automated task (reading the word). The difference between WCN and CN is considered an expression of the Interference component (77).

#### Multiple-choice task

MCT assess selective attention. The target can be a number (1, 2, 3) whose presentation is randomized. Each number can be associated to a different response button. In each trial patients have to press as quickly as possible the response button associated with the number that appeared on the screen. The accuracy of responses is evaluated by counting the errors (56, 75).

For each task, the mean value can be computed and RTs distributions can be fit with ex-Gaussian distribution using maximum likelihood estimation ad a bounded Simplex algorithm (78). From the resulting ex-Gaussian function three parameters, μ, σ, and τ can be obtained: the first two parameters (μ and σ) correspond to the mean and standard deviation of the estimated Gaussian component (sensory-motor and automatic processes), the third parameter (τ) is the mean of the estimated exponential component (central, attentive and decision-related processes of executive attention).

### Application of TMS

We intend to adopt TMS for understanding motor cortex excitability and the functioning of intracortical circuits in fatigued and non-fatigued PD patients, to carry on a neurophysiological evaluation of motor fatigue and to evaluate possible relations with mental-cognitive fatigue. Therefore, the following measures will be collected:

#### Resting motor threshold

RMT is defined as the lowest TMS intensity (expressed in percentage of the maximum stimulator output) that evoked MEPs of at least 50 μV peak-to-peak amplitude in five of ten successive trials (79).

#### Cortical silent period

CSP reflects an intracortical, mainly GABA_B_-mediated inhibitory phenomenon, and is defined as the time elapsing from the end of the MEP until the recurrence of voluntary tonic electromyographic activity (79).

#### Short and long interval intracortical inhibition

SICI is thought to represent GABA_A_-receptor-mediated fast inhibitory post-synaptic potentials (IPSPs) in corticospinal neurons, while LICI is considered a phenomenon dependent on slow IPSPs mediated through GABA_B_-receptors (80).

#### Short-latency afferent inhibition

SAI is a marker of inhibitory sensorimotor integration that depends mainly on the excitatory effect of cholinergic thalamocortical projections onto the inhibitory GABAergic cortical network (81).

#### TMS evaluation of neuromuscular fatigue

Neuromuscular fatigue is typically assessed via sustained isometric maximal voluntary contraction (82). MEP amplitude and CSP duration can be evaluated to assess neuromuscular fatigue 10 min before (PRE) and 2 min after (POST) a 1-min fatiguing motor task. After a fatiguing isometric exercise, MEPs evoked in the resting target muscle are depressed for about half an hour. The CSP, on the opposite, increases after a fatiguing isometric muscle effort likely with the physiological purpose to reduce corticomotor output and prevent excessive peripheral exhaustion (83).

## Conclusions

To the best of our knowledge, no studies have investigated IIV in parkinsonian subjects with respect to their level of fatigue and no data examining the relations between IIV in RTs and TMS measures actually exist. Combining these measures and correlating the results of neuropsychological investigations with the neurophysiological ones could help in the attempt to understand whether, and to what extent, alterations of the attentional system contribute to the perception of physical and mental fatigue in Parkinsonian patients. Therefore, results from further studies adopting these neuropsychological and neurophysiological measures and based on this framework could help in understanding physical and cognitive fatigability in PD.

## Author contributions

PO, DF, AR, JS, and SD substantial contributions to the paper conception. PO, DF, and NG drafting the work. VV, LSa, RM, SB, AO, and LSe revising the paper critically for important intellectual content. All authors contributed to the article and approved the submitted version.
